# Online information and availability of three doping substances (anabolic agents) in sports: role of pharmacies

**DOI:** 10.3389/fphar.2023.1305080

**Published:** 2023-12-04

**Authors:** Juan F. Garcia, Jesús Seco-Calvo, Soledad Arribalzaga, Raquel Díez, Cristina Lopez, M. Nelida Fernandez, Juan J. Garcia, M. Jose Diez, Raul de la Puente, Matilde Sierra, Ana M. Sahagún

**Affiliations:** ^1^ Department of Mechanical, Informatics, and Aerospatiale Engineering, University of Leon, Leon, Spain; ^2^ Physiotherapy Department, Institute of Biomedicine (IBIOMED), University of Leon, Leon, Spain; ^3^ Psychology Department, Faculty of Medicine, Basque Country University, Leioa, Spain; ^4^ Department of Biomedical Sciences, Institute of Biomedicine (IBIOMED), University of Leon, Leon, Spain

**Keywords:** androstenedione, athletes, dehydroepiandrosterone (DHEA), doping substances, internet availability, internet sale, oxandrolone

## Abstract

**Background:** The Internet has become an important source for easy access to doping substances, where people and athletes may acquire, outside pharmacies and without a (medical) prescription. These online websites do not always offer quality-assured products, and are outside the regular distribution channels of medicines. The aim of this study was to estimate the availability and accessible information on the Internet about the sale of three doping substances (oxandrolone, DHEA, androstenedione).

**Methods:** Cross-sectional exploratory study, being an observation at a point in time of the online availability of these three doping substances (WADA S1 category: anabolic agents), purchased from Spain, Puerto Rico, Canada, United States, Ukraine and Russia. The characteristics of the websites, the countries the webs sold to, the pharmaceutical forms offered and the recommendations for its use were analyzed by using a computer tool designed *ad hoc*.

**Results:** There were significant differences between countries in the number of webpages that sold the products (Chi-square test, *p* < 0.05). Oxandrolone was available for purchase mainly when buying from Spain (27.12%) and Ukraine (26.58%), in websites dedicated to sports (77.26%). For DHEA, most of the pages offered it if the search was done from Canada (23.34%) and Russia (21.44%). Products containing androstenedione or DHEA are claimed to enhance sports performance or for sports use without providing details. Compared to the total number of websites checked, the proportion of pharmacies offering these products was low, ranging from 4.86% for DHEA to 15.79% for androstenedione.

**Conclusion:** The three substances selected are easily available without control through the Internet. Only a small number of websites offering them were online pharmacies, and requested a prescription. Most of the doping substances are purchased from the country where they are requested. Product information described benefits for sports performance, but did not do the same with their side effects. It would be advisable for these products to be sold through pharmacies, to guarantee their quality and provide evidence-based information on their safe use, benefits and risks, and only with a prescription. Athletes should be encouraged to consult health professionals about those supplements suitable for their type of training and sports objectives.

## 1 Introduction

The use of doping substances among athletes has increased in recent years (Martínez-Sanz et al., 2017; Sellami et al., 2018; Wardenaar and Hoggervorst, 2022). Their use makes it possible to cover deficiencies derived from ingestion, but also to optimize exercise performance and improve body composition and recovery processes (Birzniece, 2015; Knapik et al., 2016; Savino et al., 2019; Sadek et al., 2022). The latter are essential in athletes due to the increased physical demands (Stellingwerff, 2013).

On the other hand, the World Anti-Doping Agency (WADA) defines doping as: “the commission of one or more anti-doping rule violations as provided for in sections 1–11 of Article 2 of the Code” (Garthe and Maughan, 2018). These sections mention the concept of prohibited substances, metabolites or markers that may be present in the athlete’s sample. These substances are included in the Prohibited List of the World Anti-Doping Code. The requirements to be incorporated into this category are to accomplish two of three criteria: 1. it has the potential to optimize performance; 2. it has an actual or potential risk to the athlete’s health; 3. it violates the competitive spirit as expressed in the Code (World Anti-Doping Agency, 2021).

Steroid drugs commonly used as androgens and anabolic agents that increase endogenous testosterone levels are divided into 3 groups: natural steroids, synthetic ones, and drugs that increase endogenous testosterone (indirect doping). Their androgenic effects on athletic performance were confirmed from research, showing that testosterone increased muscle mass and strength. The benefits were seen without exercise, with gains in muscle mass and strength by 10%, and also with exercise, being the increases between 20% and 37% (Bhasin et al., 1996). These compounds are also been used therapeutically to treat a variety of medical conditions.

For this study, we have selected 3 substances that act as anabolic agents, two are natural steroids and testosterone precursors (pro-androgens), androstenedione and dehydroepiandrosterone (DHEA), and one is a synthetic steroid, oxandrolone. Natural androgen precursors like DHEA and androstenedione are converted to testosterone or dihydrotestosterone (DHT) or epitestosterone, and also used to mask the testosterone/epitestosterone ratio (Miller, 2020).

Oxandrolone, dehydroepiandrosterone (DHEA) and androstenedione are considered by WADA as doping substances in sports. Specifically, these substances are on the list of prohibitions that came into force in January 2023. They are included in the section of prohibited substances and methods at all times (in- and out-of-competition), in category S1 anabolic agents (World Anti-Doping Agency, 2022b).

The clinical experience of some authors of this paper as sports physicians shows that oxandrolone, DHEA and androstenedione are substances in great demand within sports environment, particularly in sports where is necessary to develop strength and gain muscle mass. Therefore, their use is common in sports such as weightlifting, bodybuilding and endurance sports. Anabolic-androgenic steroids (AAS) use is 11% among gym athletes, 39% among bodybuilders and 67% among weightlifters (Birzniece, 2015).

Regarding oxandrolone, it is an anabolic steroid synthesized in 1962 (Guddat et al., 2013). This drug is highly anabolic, and only a few androgenic effects are observed (Kuhn, 2002; Guddat et al., 2013). Its use is approved for the preservation of lean tissue in catabolic conditions, such as sarcopenia (Mavros et al., 2015). It seems to be the most popular anabolic-androgenic agent among women due to these weak androgenic effects (Ip et al., 2010; La Vignera et al., 2018), and it is among the most commonly used AAS (Piacentino et al., 2015; Smit and de Ronde, 2018; Pereira et al., 2019; Whitaker et al., 2021; Escalante et al., 2023).

In the case of DHEA, it is an androgen precursor, commonly used in premenopausal and postmenopausal women as hormone therapy (Rabijewski et al., 2020). In sports its use is justified due to the effects on body composition, decrease in fat mass and increase in muscle mass (Collomp et al., 2015). DHEA has been aggressively marketed as it increases testosterone levels and muscle mass (Brown et al., 2006).

Androstenedione is a steroid hormone intermediate in the production of estrogen and testosterone. Due to its relevance in the intrinsic synthetic pathways of androgens, it is used among athletes to achieve a change in body composition, whether it is a decrease in fat mass or an increase in muscle mass (Tokish et al., 2004). On the other hand, the favorable anabolic effects of androstenedione are inconclusive (Rasmussen et al., 2000; Brown et al., 2006), but the adverse effects such as reduced sperm count, impotence, and gynecomastia are conclusive (Badawy et al., 2021).

Regardless of the benefits reported in sports, the use of these doping substances carries health risks which involved all organs and body functions, and especially long term, when causing cardiovascular and reproductive toxicity (Labrie et al., 2006; Albano et al., 2021). There are still those who try and acquire these substances due to their androgenic effects, being unaware or disregarding their harmful effects. In this sense, the Internet has become an important source for easy access to AAS worldwide (Cordaro et al., 2011), not always within the legal channels of purchase. It is also used as a major source of information, but sometimes websites are not entirely reliable or may even be misleading and deceptive (Brennan et al., 2016).

In view of this, the aim of this study is to estimate the availability and accessible information on the Internet about the sale of these three doping substances, oxandrolone, DHEA and androstenedione, as well as to assess the labeling of the products containing each of these substances, reviewing their indications for use included in these labels. Secondarily we will estimate the offer of these compounds when buying from different countries, which would mean that there is greater tolerance to its sale depending on the State and its regulations.

It is known to the authors that there is no research on doping substances and their acquisition through Internet sites. In previous research, the analysis of doping substances is focused on the motivations for their consumption (Petróczi, 2007; Morente-Sánchez and Zabala, 2013) and/or on the advice given by trainers (Zmuda Palka et al., 2023) or pharmacists (Malek et al., 2014; Howard et al., 2018). Our hypothesis is that athletes can acquire these doping substances in certain countries without a (medical) prescription, and from websites that do not always come from laboratories.

## 2 Material and methods

### 2.1 Design

Cross-sectional study, being an observation at a point in time of the online availability of the three compounds selected that can be used as doping substances (oxandrolone, DHEA, and androstendione), and bought from six countries (Spain, Puerto Rico, Canada, United States, Ukraine and Russia). These countries were selected for several reasons: some of them were monitored by WADA in October 2022 (Russia, Ukraine, and Puerto Rico) (World Anti-Doping Agency, 2022a); two other countries as they host the Compliance Review Committee (CRC) and the International Federation of Bodybuilding and Fitness (IFBB) (Canada and Spain, respectively); and the United States of America, due to the number of athletes (Barrack et al., 2020; USADA, 2023) and the annual revenue from the sale of sports supplements, with more than 1.4 billion in annual revenue (Badawy et al., 2021). Thus, we decided to analyze these supplements because they may be frequently consumed by athletes, and their intake is often associated with improvements in sports performance, either by increasing muscle mass (Jenkinson and Harbert, 2008), their effects on sex hormones (Mason et al., 2001) or their anti-inflammatory and antioxidant actions (Cerqueira et al., 2020).

To carry out the searches, an “*ad hoc*” software was used and programmed. By using the computer tool designed, it was simulated to be a purchaser of these products from each of the countries described above. The results obtained in the different searches were reviewed and evaluated by pairs. Any potential disagreement was resolved by all authors. The searches and data evaluation took 15 days (from 1st to 15th July 2023).

The Strengthening the Reporting of Observational Studies in Epidemiology (STROBE) Statement was used to report data (Cuschieri, 2019) and the checklist included in the Supplementary Material (see Appendix A).

### 2.2 Data obtained from the website pages analyzed

The following characteristics of the websites found were assessed: number of pages that did not connect, number of pages that sold that compound, number of pages that sold any other of the compounds evaluated and which of them; type of page, that is, if it was a specific sports page, a supermarket, a pharmacy or a parapharmacy, if it was specialized in the sale of nutritional supplements, a laboratory, pages that sold any type of product, or any other type of page. In addition, it was checked whether they sold only within their own country, only to several countries or globally. For each of the three compounds studied, we also evaluated the pharmaceutical forms offered, e.g., whether they were for oral, parenteral or other routes of administration; what the product was recommended for; and whether any type of prescription was required for purchase.

### 2.3 Search engine

To gather all the information we needed from the Internet, we used our previous experience, adapting our software and masking our global location using a VPN.

We gathered seeds using a modified version of our crawler and IMN Search Engine Extractor v32 (García and Carriegos, 2020), adapted from our previous researches (García et al., 2020; García et al., 2022), choosing Google as our search engine. Our crawler can automatically visit a list of predefined websites (the aforementioned seeds), extracting and (or) analyzing information they contain, which greatly eases the whole process (we do not have to manually search for and visit sites), reducing fatigue and propensity for human error.

The keywords used to obtain these seeds were “oxandrolone”, “dehydroepiandrosterone”, “DHEA”, and “androstenedione”, translated to the languages spoken in each of the regions we were interested in (Spanish, English, Russian, and Ukrainian). Our software takes the list of URLs to visit directly from the search engine’s (that is, Google) result page (SERP).

In this research, we found two major inconveniences to overcome: mandatory continuous scrolling SERP’s configuration and (also mandatory) omitted related results.

On the one hand, we found problems when working with Google regions in which continuous scroll search (a new feature from Google active since April 2023) was available (United States). Results in those areas are loaded in batches, ten by ten, while the user scrolls down; when reaching one hundred results, the whole search results page reloads, and the user must start scrolling again (the same results) although this time more results are offered (typically, around two hundred max). Even if the search engine can be set up so continuous scroll is deactivated, it does not work, and the option to set “maximum results per page” is overridden by this setup (as stated by Google itself). All this makes search extremely costly to automate (we do not get all results in one click, we have to scroll for them to load), and we had to rely on Selenium to do so, simulating the user scrolling down to the bottom of the SERP until no more results were loaded.

On the other hand, SERP showing (initially omitted) related results is no longer an option, but an automatic action once last page of results (or last batch of results - for continuous scrolling regions - and yes, this problem goes on top of the previously detailed one for that region) is loaded in the browser. In our experience, loading related results is almost never useful (Afterall, Google itself had omitted them for a reason): most (if not all) of them are duplicated, or pertain to the same domain of other results, and are thus irrelevant. Showing related results artificially boosts results offered by the engine from (usually) two to up to five times the number of original results, so a little bellow one hundred quickly becomes several hundred; however, as expected, these results were highly undesired, and we did not find a fix for this behavior (since it is on Google’s side and now it is a compulsory functionality of the search engine), so we had to make additional scripts and software changes to wait for them to load and then purge the results, limiting seeds to one by domain.

As for the VPN to use, we checked several alternatives: ExpressVPN, NordVPN, Surfshark (all of them requiring payment, although ExpressVPN has a free trial period), Tunnel Bear, Urban VPN, or Windscribe (offering a free subscription), just to name a few. Tunnel Bear is a decent and free VPN popular within the Internet community, especially in Reddit forums. It offers servers in 49 countries and comes with 500 MB/mo bandwidth. It provides a wide server network, military-grade encryption, and a zero-log policy, making a decent free VPN. Windscribe is another interesting alternative, offering higher bandwidth (10 GB/month) but fewer world servers (10 countries). ExpressVPN and NordVPN are more robust, limit-free, and reliable (major drawback with free options is that they either sell your data or bloat you with adds - or both) options, as long as you pay for the subscription.

We had to discard free options since neither of them included all the servers we were interested in their free subscription, but Windscribe does for their Pro (paid) version. Among the payed options we checked, NordVPN offered all the countries we were looking for, and Urban VPN (free) offered all but one (Puerto Rico), so we went for the latter. To get Puerto Rico’s data, we used Google’s region option, which is less representative of the real scenario: Google’s offering country-specific results to someone really being at the country since he/she is using a local ISP *versus* Google offering country-specific because you ask it for; in the first case, is on their own interest to do so (so they will probably do it to the best they can), while on the other hand it is just a user’s request. Even if that’s the case, we found results obtained to be reliable and useful for our research in this scenario.

### 2.4 Statistical analysis

All information was collected in a Microsoft Excel 2016 and statistical analysis was carried out using SPSS version 24. The results obtained were expressed as frequencies and percentages. The Chi-square test was performed to compare the number of websites found after carrying out the searches of the three products evaluated in the different countries, considering their characteristics and the countries they shipped to. *p* ≤ 0.05 was taken as the level of significance.

## 3 Results

Tables 1, 2 and Table 3 include the analysis of the websites found in which oxandrolone, DHEA, and androstenedione, respectively, may be acquired from the different countries. After the search, the software designed for the study located a total of 2721 websites potentially selling the compounds selected, with similar values for each of the 3 products. Subsequently, the pages were reviewed and those that either did not connect or did not sell the product were eliminated (most of them were pages with information about the product or pages of laboratories that made analytical determinations of the compound). Finally, there were a total of 977 pages that did sell any of the compounds evaluated.

**TABLE 1:** Analysis of websites found in which oxandrolone may be acquired from the different countries.

		Spain	Puerto Rico	Canada	United States	Russia	Ukraine
**Characteristics of the web pages reviewed**	**Number of web pages reviewed**	190	207	102	115	115	131
	**Do not connect** ^*^	25^b^	62^a^	45^a^	26^b^	52^a^	9^b^
	**Do not sell the product**	66	86	37	50	13	24
	Sell the product*	99^b^	59 ^a, c^	20^a^	39 ^c, d^	50 ^b, d^	98
	Also sell other products*	16	18	3	9	2	4
	**DHEA**	10	10		6	1	2
	**Androstenedione**	1		3	3		0
**Type of web pages selling the product**	**Sports**	60	51	8	28	44	91
	**Supermarket**						
	**Pharmacy**	2	3	6	2	3	3
	**Parapharmacy**	8					
	**Dietary supplements**	19	1		2		
	**Laboratory**	5	3	5	2	1	2
	**Store**	5	1	1	5	2	2
**Shipping countries**	**Only the own country**	48	24	12	4	40	94
	**Worldwide**	42	21	6	33	3	3
	**Several countries**	9	14	2	3	7	1
**Prescription required**				3	5		
**Route of administration**	**Oral**	99	59	18	36	50	97
	**Parenteral**		1				2
	**Other**			2	3		1
**Recommended use of the product** ^a^	**1**	92	48	12	35	49	81
	**2**	2	22	4	18	5	21
	**3**		8	2	16	2	8
	**4**	5	7	3	4	2	2
	**5**		3	3	1	3	4
	**6**			2	3		
	**No description**	6	9				15

*Significant differences (Chi-square test, *p* < 0.05). Each letter of the superscript denotes a subset whose proportions do not differ significantly from each other (*p* < 0.05).

^a^ 1. Sport: increase in muscle mass; increased strength, endurance and performance; 2. Burns fat (to lose body fat); 3. Hormonal activity: increased growth hormone; 4. Increased appetite, weight gain in some pathologies; 5. Relief of bone pain, improves wellbeing; 6. Research.

**TABLE 2:** Analysis of websites found in which DHEA may be acquired from the different countries.

		Spain	Puerto Rico	Canada	United States	Russia	Ukraine
**Characteristics of the web pages reviewed**	**Number of web pages reviewed**	151	86	263	147	172	116
	Do not connect*	14^b^	7^b^	37^a^	8^b^	38^a^	26^a^
	**Do not sell the product**	55	28	92	29	15	20
	Sell the product*	82 ^a, b^	50 ^a, b, c^	124^a^	110 ^d^	119 ^c, d^	70 ^b, c^
	Also sell other products*	61 ^b, c^	37 ^b, c^	79^a^	73^b^	58 ^a, c^	45 ^a, b, c^
	**Oxandrolone**	4	7	11	5	3	2
	**Androstenedione**				1	1	
**Type of web pages selling the product**	**Sports**	5	8	12	4	65	39
	**Supermarket**	1	2	5		5	
	**Pharmacy**	3	2	13	4	2	3
	**Parapharmacy**	3	6	5		38	
	**Dietetic products**	42	19	8	77		19
	**Laboratory**			44	3		
	**Store**	28	12	17	20	9	9
	**Other**		1	20	2		
**Shipping countries**	**Only the own country**	58	26	51	61	117	69
	**Worldwide**	18	10	46	40	2	1
	**Several countries**	6	14	27	9		
**Prescription required**				2	5		
**Route of administration**	**Oral**	82	49	124	106	119	70
	**Parenteral**		1		1		1
	**Other**			1	4		2
**Recommended use of the product** ^a^	**1**	35	36	34	26	65	59
	**2**	20	24	26	24	39	48
	**3**	8	9	8	2	19	12
	**4**	34	14	44	41	15	3
	**5**	10	9	4	12	1	3
	**6**	16	16	6	4	1	3
	**No description**		5		2		10

*Significant differences (Chi-square test, *p* < 0.05). Each letter of the superscript denotes a subset whose proportions do not differ significantly from each other (*p* < 0.05).

^a^ 1.- Sport: increased physical capacity, accelerated growth of muscle mass; 2.- Sex hormones: increased sexual function, impotence, fertility treatment in women, maintaining hormonal balance; 3.- regulation of the immune system, reduction of inflammation; 4.- Anti-aging, improvement of memory, mood and sleep; 5.- Improvement of health: cardiovascular, bone, nervous system; 6.- promotes metabolism, weight loss.

**TABLE 3:** Analysis of websites found in which androstenedione may be acquired from the different countries.

		Spain	Puerto Rico	Canada	United States	Russia	Ukraine
**Characteristics of the web pages reviewed**	**Number of web pages reviewed**	220	225	134	119	126	102
	Do not connect*	25^b^	25^b^	12^a,b^	5^a^	17 ^b^	11^a,b^
	**Do not sell the product**	192	197	109	93	99	84
	Sell the product*	3^c^	3^c^	13^a,b^	21^a^	10^b^	7^b^
	**Also sell other products**	3	2	10	18	6	4
	**Oxandrolone**	1		10	17		2
	**DHEA**	1			16	6	5
**Type of web pages selling the product**	**Sports**	1		1	3	2	2
	**Supermarket**						
	**Pharmacy**	1	2			4	2
	**Parapharmacy**					1	
	**Dietary supplements**					1	1
	**Laboratory**	1	1	12	17	2	
	**Store**				1		2
**Shipping countries**	**Only the own country**	1	2	1		8	7
	**Worldwide**	2	1	12	21	2	
	**Several countries**						
**Prescription required**							
**Route of administration**	**Oral**	2	2	1	3	8	7
	**Parenteral**						
	**Other**	1	1	12	18	2	
**Recommended use of the product** ^a^	**1**	2	1	1	1	6	6
	**2**	1	^a^1		3	4	6
	**3**		1	12			
	**No description**				14		1

*Significant differences (Chi-square test, *p* < 0.05). Each letter of the superscript denotes a subset whose proportions do not differ significantly from each other (*p* < 0.05).

^a^ 1.- Sport: increase muscle mass and strength; 2.- increase testosterone levels; 3.- research.

A total of 860 websites were found for oxandrolone (Table 1). The countries that most often offered the product for sale were Puerto Rico (24.07%) and Spain (22.09%). However, once the websites that did not connect or did not sell this compound (57.56% of the pages) were eliminated, the countries with the highest number of pages available for purchase were Spain and Ukraine (27.12% and 26.85% of the websites selling oxandrolone, respectively). Statistical analysis revealed significant differences between countries (Chi-square test, *p* < 0.05), but not between the following pairs: Puerto Rico and Canada; Spain and Russia; Puerto Rico and United States; United States and Russia. Regarding the type of site where oxandrolone could be purchased, the majority (77.26%) were sites dedicated to sports (sport stores or sites specialized solely in nutrition or sport pharmacology). This percentage was the highest when searching from Ukraine (92.86%) and the lowest from Canada (40.00%).

Most of the sites sent the order only to their own country (60.82%). For web pages located from Russia and Ukraine this percentage was much higher (80.00% and 95.92%, respectively) and the lowest value corresponded to sites found from the United States (10.26%). Most webs from the United States served worldwide (84.62% of the sites). Only 3 sites in Canada and 5 in the United States asked for a prescription to be able to buy it. Practically all the products offered were for oral administration. A small number of webpages from Puerto Rico and Ukraine sold oxandrolone for parenteral administration and in Canada, United States and Ukraine, in the form of a powdered chemical product. As for oxandrolone indications, most websites (86.85%) recommended its use for sports, stating that it increased muscle mass, strength, endurance and performance in sports. Oxandrolone could hardly purchase in pharmacies (only 5.20% of those webpages selling this compound), and 2.19% required a prescription.

As mentioned above, Table 2 summarizes the data obtained for DHEA, for which a total of 935 pages were found. Most of them were located by simulating the search from Canada (28.13%) and Russia (18.40%). After reviewing these websites, we found that 59.36% of them sold the compound, and the countries from which the largest number of pages were available for purchase were also Canada and Russia (22.34% and 21.44% of the websites, respectively). In this case, there were also significant differences between countries (Chi-square test, *p* < 0.05), with similar proportions among the subgroups Spain, Puerto Rico and Canada; Spain, Puerto Rico and Ukraine; Puerto Rico and Ukraine; United States and Russia. Of the total number of websites offering DHEA, 29.73% were dedicated to the sale of dietetic products and 23.96% to sports. However, if each country was evaluated independently, there were differences between them. In Spain, Puerto Rico and the United States, those dedicated to dietetic products predominated (51.22%, 38.00% and 70.00%), while in Russia and Ukraine the majority of sites were dedicated to sports (54.72% and 55.71%, respectively) and in Canada they were mainly laboratories (35.48%). There were specific cases of other types of sites, such as aesthetic clinics or veterinary clinics. As for where the orders were sent, most of the sites only sent them to their own country (68.83%), with the highest percentages, once again, corresponding to sites located in Russia (98.32%) and Ukraine (98.57%). Only 2 sites from Canada and 5 from the United States required a prescription to purchase it. Practically all the websites offering DHEA were for oral administration, only 3 pages (1 from Puerto Rico, 1 from United States and 1 from Ukraine) offered products for parenteral administration and a small percentage (4.24%) sold formulations for topical administration. The indications for this compound, as reported by the different web pages, were very varied, highlighting that it was useful for sports (45.95% of the web pages), for the improvement of disorders related to sexual hormones (32.61%) and as an anti-aging product (27.21%). In the different countries evaluated, these indications were similar. Again, the proportion of pharmacies dispensing DHEA was low (4.86%), whereas a prescription was required in only 1.26% of websites.

In the case of androstenedione (Table 3), 926 pages were found, most of them when buying from Puerto Rico (24.30%) and Spain (23.76%), but only 57 of them offered this product for sale. In this case, the vast majority of the websites found by the software carried out analytical determinations of this compound in biological samples. The countries from which there were more possibilities to acquire this product were the United States and Canada (36.84% and 22.81% of those websites selling androstenedione, respectively). After carrying out the statistical analysis, there were significant differences between countries (Chi-square test, *p* < 0.05), but not for the subgroups Canada and United States; Canada, Russia and Ukraine; Spain and Puerto Rico. A 4.64% of the webs also offered one of the other products. This compound was mostly sold by pages belonging to laboratories (33.33%). In Puerto Rico and Russia, it was mainly offered on pharmacy websites (66.67% and 40.00% of the sites, respectively). For this compound, most websites that sold it shipped worldwide (66.67%), except for those located in Russia and Ukraine, which only shipped it within their own country (80% and 100%, respectively). None of the websites selling androstenedione required a prescription to purchase it. Most of the products offered (57.90% of the websites) were the chemical compound itself in powder form (mainly Canadian and United States websites), and in the rest of the pages, it was available for oral administration. None of the sites offered parenteral formulations. Androstenedione powder was intended for research purposes, and the rest of the products offered were recommended to increase muscle mass and strength (29.82% of the sites) or testosterone levels (26.32%). The proportion of online pharmacies where this compound could be purchased was the highest of the three substances assessed (15.79%). In addition, none of the websites offering this product requested a prescription.

## 4 Discussion

In this study we have evaluated the availability and accessible information on the internet about the sale of oxandrolone, DHEA and androstenedione. It was hypothesized that athletes can acquire these doping products on the internet from pages that do not always come from accredited pharmaceutical laboratories, as well as from countries that do not always require a prescription. The variables that were analyzed allowed us to know; 1. The availability of acquiring doping substances in the same country or in other countries; 2. The type of web page; 3. The information with which these substances are marketed, e.g., administration forms and recommendations for their use.

The consumption of doping substances seeks to improve some of the four main dimensions of sports –skill, strength, endurance and recovery-. A substance or the combination of several substances will be consumed according to the dimension to be improved. In the case of the doping substances under study, their consumption would benefit strength and sports where it predominates (e.g.,; lifting, throwing, boxing, sprinting). Thus, it can be considered a direct or indirect androgenic doping. In the first case, natural androgens such as nandrolone or proandrogens (androstenedione, DHEA) would be found (Handelsman, 2000).

In the case of the substances tested, oxandrolone, a synthetic analog of testosterone, was mostly available on sports-oriented Internet sites, which shipped the order mainly in the country itself. Only 8 out of 977 sites requested a prescription. Its main form of administration was oral and, to a lesser extent, as powder (Canada, United States and Ukraine) or parenteral forms (Puerto Rico and Ukraine). 77.26% of the sites marketing this product were dedicated to sports or for purposes such as sports nutrition and pharmacology. Regarding the information label, it is described as increasing muscle mass, strength and endurance. Oxandrolone is used to preserve or restore muscle mass (Bianchi and Marbini, 2015), often in the treatment of catabolic disorders such as Duchenne’s dystrophy (Fenichel et al., 2001), injuries associated with loss of muscle mass and strength (Przkora et al., 2007; Mavros et al., 2015), improving strength (Schroeder et al., 2003) and exercise tolerance (Tamaki et al., 2001). Therefore, the information provided with the product is not wrong; the explanation behind these benefits is the stimulating action on the protein synthesis of peripheral myelin. This is the reason because oxandrolone is used in demyelinating lesions and diseases (Melcangi and Mensah-Nyagan, 2006). It should be noted that, in spite of these indications, the vast majority of webpages offering this compound were neither pharmacies (94.8%) nor did they require a prescription (97.8%), with the severe consequences that its consumption may have on the health of athletes, professional or amateur.

As for DHEA, it is a product widely available on the Internet, having been found to be actually sold on 555 websites. These sites were dedicated to the sale of dietary products (29.73%) or related to sports (23.96%). In addition, they offered some of the other two substances, and their recommendations were diverse, mainly sports use (45.95%), benefits in disorders associated with sex hormones (32.61%) and anxiety (27.21%). Its administration was mainly oral, followed by parenteral and topical. Again, the number of pharmacies dispensing this product was low (4.9%), and only 7 websites required a prescription (1.3%), which gives an idea on the ease of access to this substance without recognized professional advice and the harmful effects that this use may have for the consumers.

Its inclusion in WADA’s list of prohibited substances is due to performance enhancements as it increases testosterone (Collomp et al., 2015; Collomp et al., 2018; Gravisse et al., 2019). DHEA administration is sometimes combined with testosterone and its precursors such as androstenedione (Alkatib et al., 2009; de la Torre et al., 2019).

The results for androstenedione showed, on the one hand, that the possibility of acquiring this substance through the Internet is much lower than for the other two compounds, and most webpages found were dedicated to determine this product in analytics. On the other hand, the main countries where it was offered were United States and Canada. Particularly, in the United States, consumption of androstenedione has become popular since a baseball league player admitted to using it (Badawy et al., 2021). Likewise, in the case of this doping substance, none of the Internet sites required a prescription. It should be noted that androstenedione is frequently prescribed in sarcopenia in older adults for the purpose of improving quality of life (Brown et al., 2000). Since it is a precursor of testosterone, its consumption facilitates the increase of muscle mass as well as the performance in the training sessions (Badawy et al., 2021). It is sold as an anabolic agent (Catlin et al., 2002) and used as a supplement for bodybuilding to improve performance (Badawy et al., 2021). In the results, it was observed that recommendations for its consumption are associated with enhancing muscle mass and strength (29.82%) or increasing testosterone levels (26.32%). Androstenedione, unlike oxandrolone, was mainly marketed in laboratories (33.33%) and pharmacies (66.67% in Puerto Rico and 40% in Russia). This compound was mainly offered as active ingredient (powder) (57.90%) and secondly for the oral route.

As described before, AAS therapies are indicated in disorders associated with aging, cachexia, cancer or osteoporosis (Kanayama et al., 2010). However, some athletes consume AAS to fraudulently achieve an increase in protein synthesis and thus in muscle mass and strength, as well as reduce body fat (Hengevoss et al., 2015). High doses of AAS may lead to physical complications such as hypertension, abnormal blood clotting, hepatotoxicity and hepatic tumors, tendon damage, reduced libido, impaired glucose tolerance, increased blood cholesterol and psychiatric/behavioral symptoms (Shephard et al., 1977; Finkelstein et al., 2013; Piacentino et al., 2015). Regarding mood disorders, hospitalizations for manic episodes (Papazisis et al., 2007) and suicides (Pärssinen et al., 2000) have been reported, both associated with long-term consumption. When athletes choose to take them, they sometimes do so without medical supervision. This situation, coupled with the easy access to these substances without a medical prescription through the Internet, and the scarce or distorted information provided by commercial websites, may aggravate the adverse reactions described above.

Regarding the status of the tested substances in the countries evaluated, it may change from one country to another, as a compound may be approved for use in one country and not in another. Nevertheless, and although WADA includes these three substances in the list of prohibited substances for athletes at all times (in- and out-of competition) (World Anti-Doping Agency, 2022b), we have observed that recommendations of use are focused frequently on sport improvements.

The six countries evaluated in this study are adhered to the WADA Convention against Doping in Sport, and comply with guidelines and the lists of prohibited substances established by World Anti-Doping Agency, 2022b. However, in the case of Russia, the meetings held by WADA in September 2023 persist in the analysis on the reinstatement of the Russian Anti-Doping Agency (RUSADA) because, even after the deadline provided by WADA, the RUSADA agency does not comply with these conditions and remains on the Global List of Non-Compliant Signatories and Applicable Consequences (World Anti-Doping Agency, 2023).

The problem underlying this supply and easy access to substances through the Internet is that people, athletes or not, are not always able to distinguish whether this consumption is legal or not. It would be simpler if they were only accessed through regular distribution channels of medicines like the pharmacies. If offered as medicines, these substances should require a prescription, given their steroidal nature, and certainly they should not be offered as supplements. As for DHEA, although in many countries this steroid is under legal control, in the United States it is not included in the Controlled Substances Act, and it is also allowed in dietary supplements. Conversely, DHEA is listed as a controlled substance by Health Canada because of its effect on hormones, and need a prescription, and in the European Union it could also not be used as a supplement. In any case, it should be noted that if athletes from all these countries participate in international competitions, they have to comply with the World Anti-Doping Code to avoid sanctions.

Misinformation and the easy availability of these substances favor the increase in consumption. In the United States, 3.0% of young people have used doping substances, specifically anabolic-androgenic steroids (Hildebrandt et al., 2012). In addition, a 60% consumption of dietary supplements is reported by athletes (Schröder et al., 2002; Schröder et al., 2004; Barrack et al., 2020), that may lead to inadvertent doping (Helle et al., 2019; Mallick et al., 2023). Thus, another study (Badawy et al., 2021) highlighted that the main source of information at the time of purchasing supplements came from friends or the media and, to a lesser extent, from professional advice. Also, in the case of professional athletes, 28% of them had consulted a physiotherapist about their use, but 18% acknowledged that they used supplements without seeking professional support (Judge et al., 2016). There are studies (Malek et al., 2014; Howard et al., 2018) showing that pharmacists were used as a source of information in the selection and purchase of products less frequently than other information providers.

Thus, it would be important to harmonize the sale of these substances through pharmacies and not through these websites. Pharmacies are regular channels of distribution of medicines, so that it would be possible to control their sale only when they are therapeutically needed. At the same time, pharmacies may provide evidence-based information on their use and the benefits and risks associated. On the other hand, it is possible that these products offered through the Internet are falsified medical ones, possibility that would be eliminated if they were dispensed only through pharmacies.

Several studies have highlighted the need for better communication on health risks of purchasing substances online, as well as the need to establish sales control procedures (Ventola, 2014; Long et al., 2022; Qutob et al., 2023). National and international public health authorities and regulatory agencies should direct their efforts to clearly regulate the sale of the substances evaluated in this study (and others in the same situation) exclusively for the therapeutic use for which they are indicated, as well as harmonize the sale of dietary supplements. On the other hand, control measures for the sale of doping substances through the Internet should be established, with cybersecurity measures that help to monitor and control this market.

Moreover, emphasis should be placed on ergonutritional education on which supplements are allowed, but also on the proper way to select them, e.g., brands, modes of administration, timing of ingestion (Malek et al., 2014). The risks of their consumption do not only extend to a sanction by WADA, but also to adverse health effects (Badawy et al., 2021). These facts highlight the importance of professional advice before the consumption of these substances.

### 4.1 Limitations and future perspectives

This study is not without limitations. The websites described were located at a point in time, so these data should be updated on a regular basis as online offer is constantly changing. Additionally, some websites can disappear and new ones may appear. This study serves as a backbone for future research related to the proposed topic, including more prohibited substances from the same category.

## 5 Conclusion

From this study it is concluded that these substances are easily available without control on the Internet, with only a limited number of sites offering oxandrolone, DHEA and androstenedione requesting a prescription. Particularly in the case of androstenedione, none of the websites requested a prescription. Products’ information for its consumption refer to performance benefits and increases in muscle mass, strength and endurance, but they do not do the same for their side effects. Only a small number of websites selling these products were online pharmacies. It would be important for these products to be sold through pharmacies, as it would guarantee their quality and, in addition, provide evidence-based information on their safe use, benefits and risks. It has been found that, despite regulatory measures, websites are available in the countries evaluated to purchase these substances without a prescription. Most of the web pages offering these compounds only sell them in their own country, but it is possible to purchase them in some sites that send to other countries.

## 6 Practical applications

It should be considered that beyond the implications of their consumption in sport, they have clear detrimental effects (e.g., cardiovascular, hormonal imbalances). Athletes should be encouraged to consult health professionals (pharmacists, sport physicians and/or nutritionists) about which supplements are suitable for their type of training and sport objectives.

## Data Availability

The raw data supporting the conclusion of this article will be made available by the authors, without undue reservation.
